# Electromagnetic time-harmonic and static field polygonal rotator with homogeneous materials

**DOI:** 10.1038/s41598-019-51637-4

**Published:** 2019-10-22

**Authors:** Weijie Gao, Huaping Wang, Faxin Yu

**Affiliations:** 1 Institute of Marine Electronics Engineering, Zhejiang University, Hangzhou, 310058 China; 2 School of Aeronautics and Astronautics, Zhejiang University, Hangzhou, 310027 China

**Keywords:** Transformation optics, Optical manipulation and tweezers

## Abstract

We propose a scheme of designing polygonal rotator with homogenous materials by using linear coordinate transformation. Our strategy is available for both time-harmonic electromagnetic field case and static field case. In particular, we found that only one anisotropic material is needed in static field case, and the density of field in the central region can be altered to be denser or sparser, or stay the same. The magnetostatic field rotator can be realized by multilayered structure composed of ferromagnetic materials and superconductor, and the direct current rotator can be realized by metals with different conductivity. Numerical results verify the effectiveness of our strategy in both time-harmonic field case and static field case.

## Introduction

Pendry *et al*. proposed the transformation optics method to control electromagnetic field^1^. This new method pave a new way to design electromagnetic devices such as invisibility cloaks^1–11^, field concentrators^12^, electromagnetic wormholes^13^, optic black holes^14,15^, bending wave guides^16–18^, metalens^19–21^, field rotators^4,22–25^, and so on. The field rotator was first proposed by Chen *et al*.^4^ in 2007. This rotator can be divided into two parts, rotator shell and inner cylinder. The inner cylinder rotates a certain angle around the symmetry axis. When the radius approaches external radius of the rotator shell, the rotational angle is reduced to zero. However, the electromagnetic parameters of this kind of field rotator are inhomogeneous and anisotropic, which are difficult to realize. A rotator with reduced parameters were experimentally proposed to simplify the design and fabrication^22^, but imperfect rotation field performance was introduced due to impedance mismatch. Inspired by Chen’s work, versions of heat fluxes were proposed^23,25^, which are composed of inhomogeneous and anisotropic conductivity materials. As the parameters in the rotators involve inhomogeneous parameters, they are not easy to implement.

One approach to simplify the constructive parameters of rotator is the linear homogeneous coordinate transformation, which can eliminate the inhomogeneity of parameters. The linear homogeneous coordinate transformation was proposed by Xi *et al*.^26^. By transforming a large triangle to a smaller one, a homogeneous one-dimensional cloak was designed. This approach was later extended to omnidirectional polygonal cloaks^27^. The whole polygonal cloak is divided into several triangles, and in each segment a linear homogenous coordinate transformation is applied, and the cloaked region is transformed from a large area to a smaller one. A twofold spatial compression method was also proposed to design a polygonal cloak by expanding several lines or a line into a concealed region^28,29^. The linear homogenous coordinate transformation is further applied to concentrator^30^, waveguides^31^ and plasmonics^32,33^ to overcome the spatial variation.

In this paper, we use the linear coordinate transformation to design an electromagnetic field rotator which is composed of homogenous materials. We extend this strategy to static field case which can be implemented by realizable materials. We show that two kinds of anisotropic materials are needed to design a time-harmonic electromagnetic field rotator. While in static field case, we show that only one anisotropic material is needed. This anisotropic material can be realized with multilayered structure composed of two isotropic materials based on the effective medium theory. Moreover, for static field cases, the field in the central region can be rotated and at the same time be denser, sparser, or unchanged.

### Electromagnetic field rotator

**Figure 1:**
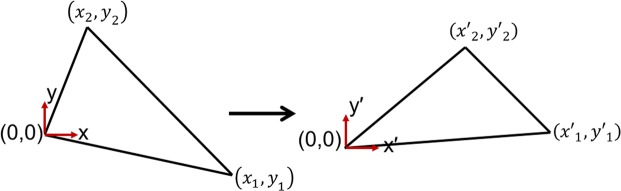
Coordinate transformation between two triangles. A triangle in virtual space (left) is transformed linearly into another triangle in physical space (right).

We firstly consider the linear transformations in two-dimensional space from a triangle in virtual space with local coordinate axes (*x*, *y*) to another triangle in physical space with local coordinate axes (*x*′, *y*′) (Fig. 1):

1 $$\begin{array}{rcl}{\rm{x}}^{\prime}  & = & {a}_{1}x+{b}_{1}y+{c}_{1}\\ y^{\prime}  & = & {a}_{2}x+{b}_{2}y+{c}_{2}\end{array},$$

Substituting coordinate of the three points in (1), we can get

2 $$\begin{array}{rcl}J & = & (\begin{array}{cc}{a}_{1} & {b}_{1}\\ {a}_{2} & {b}_{2}\end{array})=(\begin{array}{cc}{x^{\prime} }_{1} & {x^{\prime} }_{2}\\ {y^{\prime} }_{1} & {y^{\prime} }_{2}\end{array}){(\begin{array}{cc}{x}_{1} & {x}_{2}\\ {y}_{1} & {y}_{2}\end{array})}^{-1}\end{array},$$

where J is the Jacobian matrix. By using transformation optics method, we can obtain the electromagnetic parameters of the triangle in physical space, which are

3 $$\varepsilon ^{\prime} =(J\varepsilon {J}^{{\rm T}})/\det (J),\mu ^{\prime} =1/\det (J),\,{\rm{for}}\,{\rm{TM}}\,{\rm{wave}},$$

4 $$\mu ^{\prime} =(J\mu {J}^{{\rm T}})/\det (J),\varepsilon ^{\prime} =1/\det (J),\,{\rm{for}}\,{\rm{TE}}\,{\rm{wave}}.$$

Note that $\det (\varepsilon ^{\prime} )=\det (\varepsilon )$ for TM wave and $\det (\mu ^{\prime} )=\det (\mu )$ for TE wave, because $\det (J{J}^{T}/\det (J))=1$.

**Figure 2:**
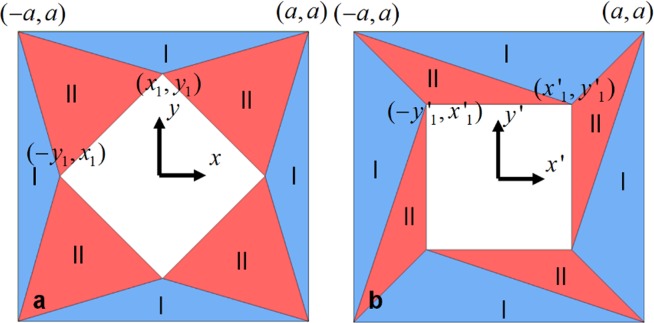
Scheme of coordinate transformation of a quadrate rotator. The rotator shell is divided into 8 segments, which can be grouped into two types (marked in blue and red, respectively). Each segment in virtual space (**a**) is transformed linearly into its corresponding segment in physical space (**b**) along its local coordinate axes.

Our strategy is dividing a polygon into several triangle segments, and then applying a linear coordinate transformation in each triangle segment. We take the quadrate rotator shown in Fig. 2 as an example to demonstrate our scheme. The coordinate system is built with the origin at the center of rotator. The rotator shell can be divided into 8 segments, which can be grouped into two types: segment I and segment II marked in blue and red, respectively. Each triangle segment in virtual space is linearly transformed to its corresponding segment in physical space. The central region is only rotated by certain degree (*θ*) without changing its sizes, which means that the constructive parameters of this area is unchanged after coordinate transformation. Compared with ref. ^24^, the transformation function in our paper is different, as a result, the rotation angle is very flexible. From Eq. (2), with a linear transformation, the Jacob matrix will be independent of the position (x, y), i.e., it will be a constant matrix. Therefore, from Eqs (3) and (4), one can see that the constitute parameters of the rotators in all regions will be independent of the position and become homogeneous. This facilitate the practical realizations.

Let’s consider the TM wave case and that all segments in virtual space are vacuum. According to the procedure mentioned above, we can get the parameters of each segment, which are

5 $${{\varepsilon }_{1}}^{^{\prime} }=({J}_{1}{{J}_{1}}^{{\rm T}})/\det ({J}_{1}),{{\mu }_{1}}^{^{\prime} }=1/\det ({J}_{1}),$$

where $${J}_{1}=(\begin{array}{cc}a & ({x^{\prime} }_{1}-{x}_{1})/({y}_{1}-a)\\ 0 & ({y^{\prime} }_{1}-a)/({y}_{1}-a)\end{array})$$, for segment I,

6 $${{\varepsilon }_{2}}^{^{\prime} }=({J}_{2}{{J}_{2}}^{{\rm T}})/\det ({J}_{2}),{{\mu }_{2}}^{^{\prime} }=1/\det ({J}_{2}),$$

where $${J}_{2}=(\begin{array}{cc}a-{y^{\prime} }_{1} & {x^{\prime} }_{1}+{y^{\prime} }_{1}\\ {x^{\prime} }_{1}-a & {y^{\prime} }_{1}-{x^{\prime} }_{1}\end{array}){(\begin{array}{cc}a-{y}_{1} & {x}_{1}+{y}_{1}\\ {x}_{1}-a & {y}_{1}-{x}_{1}\end{array})}^{-1}$$, for segment II.

**Figure 3:**
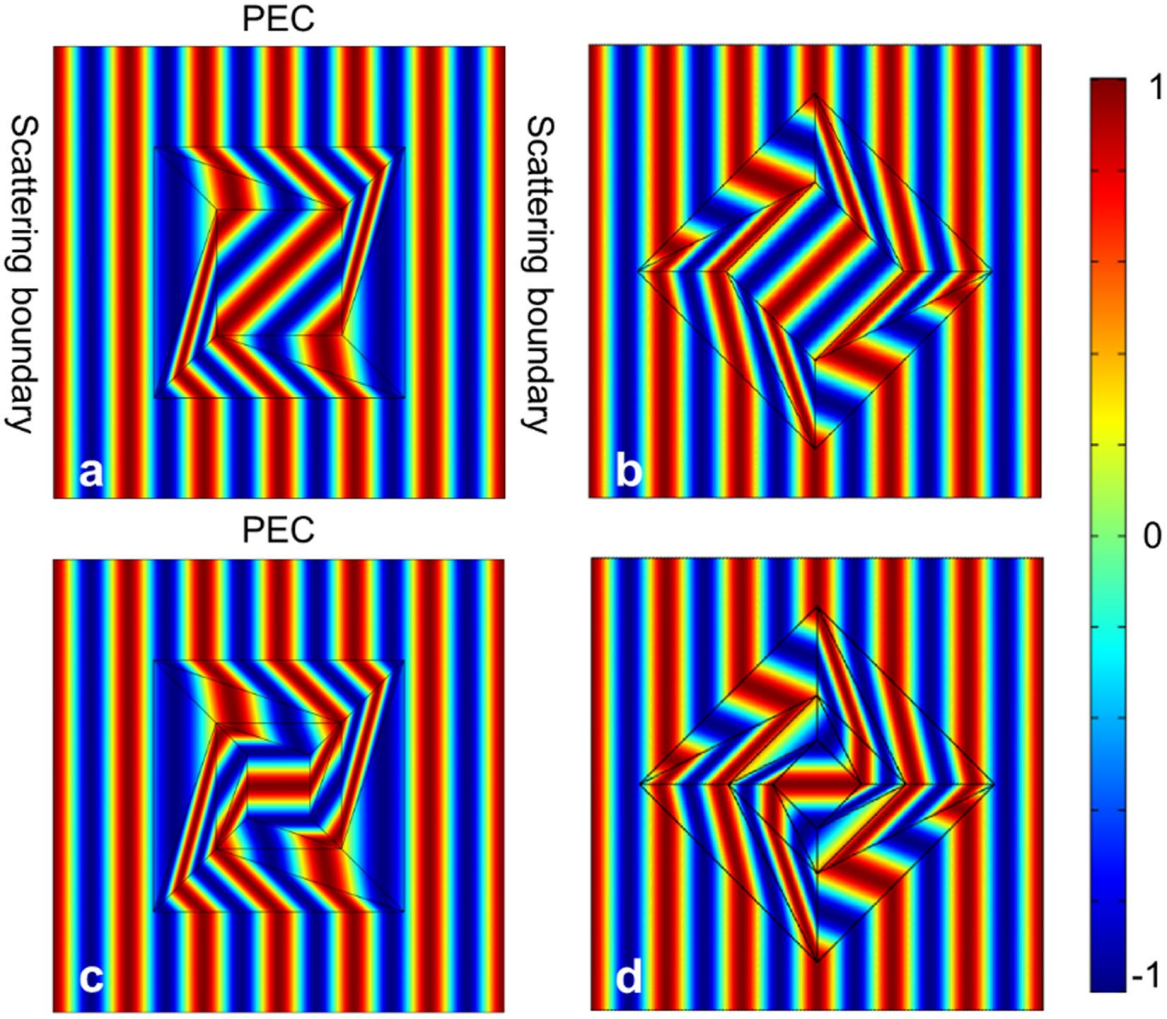
(**a**,**b**) The magnetic field distribution when an Hz polarized plane wave is incident along $\hat{x}$ direction and $\frac{\sqrt{2}}{2}\hat{x}+\frac{\sqrt{2}}{2}\hat{y}$ direction, respectively. (**c**,**d**) The magnetic field distribution of twofold rotator when an Hz polarized plane wave is incident along $\hat{x}$ direction and $a\frac{\sqrt{2}}{2}\hat{x}+\frac{\sqrt{2}}{2}\hat{y}$ direction, respectively.

In the following, we make full wave simulations by employing the commercial finite element method software, COMSOL Multiphysics, to demonstrate the performance of the rotator. We schematically illustrate the simulation in Fig. 3(a). A TM plane wave with Hz polarization (*λ* = 0.3 *m*) is imposed from left. The top and bottom boundaries of simulation region are set as perfect electric conductor (PEC), while the left and right ones are set as scattering boundaries. The parameters of electromagnetic field rotator are:

$$(a,a)=(1/2,1/2),\,(\,-\,a,a)=(\,-\,1/2,1/2),$$

$$({x}_{1},{y}_{1})=(0,\sqrt{2}/4),(\,-\,{y}_{1},{x}_{1})=(\,-\,\sqrt{2}/4,0),$$

$$({x^{\prime} }_{1},{y^{\prime} }_{1})=(1/4,1/4),(\,-\,{y^{\prime} }_{1},{x^{\prime} }_{1})=(\,-\,1/4,1/4),$$

$${{\varepsilon }_{1}}^{^{\prime} }=(\begin{array}{cc}2.2929 & -1.7071\\ -1.7071 & 1.7071\end{array}),\,{{\mu }_{1}}^{^{\prime} }=0.5858,$$

$${{\varepsilon }_{2}}^{^{\prime} }=(\begin{array}{cc}4.0157 & -1.094\\ -1.094 & 0.547\end{array}),\,{{\mu }_{2}}^{^{\prime} }=\mathrm{1.8282.}$$

The electromagnetic field in central region is rotated anticlockwise by 45°. Figure 3(a,b) show the magnetic field distribution in and near rotator, when an Hz polarized plane wave is incident along $\hat{x}$ direction and $\frac{\sqrt{2}}{2}\hat{x}+\frac{\sqrt{2}}{2}\hat{y}$ direction, respectively. It is obvious that rotator shell rotates the field in central region 45° anticlockwise, and the field out of the rotator is undistorted. Note that when the rotational angle is larger than the critical angle, for example, −69.3° in our case, both permeability and permittivity become negative, which increase the difficulty of practical implementation. However, this limitation can be easily circumvented by adding another rotator shell out or in the rotator, as shown in Fig. 3(c,d), in which there is a second smaller rotator in the central region and field in the inner core is rotated anticlockwise by 90°.

### Magnetostatic field rotator

We go a step further and extend the scheme of rotator to magnetostatic field case. The difference between static field and time-harmonic electromagnetic field is that the magnetic field and electric field are decoupled in static field. Thus we only consider the permeability of materials in magnetostatic field, which provides more degree of freedom in designing different kinds of devices and will simplify the parameters of materials. The scheme of magnetostatic field rotator is same as that of electromagnetic rotator illustrated in Fig. 2. By applying the equations in (5) and (6), we can get the parameters of the rotator,

7 $${{\mu }_{1}}^{^{\prime} }=({J}_{1}{{J}_{1}}^{{\rm T}})/\det ({J}_{1}),$$

where $${J}_{1}=(\begin{array}{cc}a & ({x^{\prime} }_{1}-{x}_{1})/({y}_{1}-a)\\ 0 & ({y^{\prime} }_{1}-a)/({y}_{1}-a)\end{array})$$, for segment I,

8 $${{\mu }_{2}}^{^{\prime} }=({J}_{2}{{J}_{2}}^{{\rm T}})/\det ({J}_{2}),$$

where $${J}_{2}=(\begin{array}{cc}a-{y^{\prime} }_{1} & {x^{\prime} }_{1}+{y^{\prime} }_{1}\\ {x^{\prime} }_{1}-a & {y^{\prime} }_{1}-{x^{\prime} }_{1}\end{array}){(\begin{array}{cc}a-{y}_{1} & {x}_{1}+{y}_{1}\\ {x}_{1}-a & {y}_{1}-{x}_{1}\end{array})}^{-1}$$, for segment II.

9 $${\rm{If}}\,{\rm{we}}\,{\rm{set}}\,{\rm{trace}}({{\mu }_{1}}^{^{\prime} })=trace({{\mu }_{2}}^{^{\prime} }),$$

and according to Eq.(4), the eigenvalue of ${{\mu }_{1}}^{^{\prime} }$ and ${{\mu }_{2}}^{^{\prime} }$ will be the same, which means that the segment I and II can be realized by the same material. As an illusion, the geometries of magnetostatic field rotator are similar to that of electromagnetic field rotator in Fig. 3(a), except that *θ* is set as unknown. The parameters of the rotator are

$$(a,a)=(1/2,1/2),(\,-\,a,a)=(\,-\,1/2,1/2),$$

$$({x}_{1},{y}_{1})=(0,\sqrt{2}/4),(\,-\,{y}_{1},{x}_{1})=(\,-\,\sqrt{2}/4,0),$$

$${({x^{\prime} }_{1}{y^{\prime} }_{1})}^{T}=(\begin{array}{cc}\cos \,\theta  & -\,\sin \,\theta \\ \sin \,\theta  & \cos \,\theta \end{array}){(0\sqrt{2}/4)}^{T},{(-{y^{\prime} }_{1}{x^{\prime} }_{1})}^{T}=(\begin{array}{cc}\cos \,\theta  & -\,\sin \,\theta \\ \sin \,\theta  & \cos \,\theta \end{array}){(-\sqrt{2}/40)}^{T},$$

By applying Eq. (9), we can get the value of *θ*, and the parameters of segment I and II,

$$\theta =-\,{39.85}^{\circ },{\mu ^{\prime} }_{1}=(\begin{array}{cc}2.1740 & -1.5470\\ -1.5470 & 1.5608\end{array}),\,{\mu ^{\prime} }_{2}=(\begin{array}{cc}3.2519 & -0.7559\\ -0.7559 & 0.4832\end{array}).$$

The eigenvalue of ${\mu ^{\prime} }_{1}$ and ${\mu ^{\prime} }_{2}$ are the same, which are ${\mu }_{a}=0.2903$ and ${\mu }_{b}={\rm{3}}.{\rm{4445}}$. This material can be realized by a multilayered structure composed of two isotropic materials. ${\mu }_{a}=0.2903$ can be achieved by using ferromagnetic materials and ${\mu }_{b}={\rm{3}}.{\rm{4445}}$ can be achieved by using dc metamaterials^34,35^. Thus our strategy manifests that only one anisotropic material is used to design a perfect magnetostatic field rotator. Besides, if the size of central square in physical space is smaller or larger than that in virtual space, the magnetic field in this area will be denser or sparser. We can still use the same anisotropic material mentioned above to design a concentrator or a sparse device.

We firstly set $a=0.5,{x^{\prime} }_{1}=0,{y^{\prime} }_{1}=t\sqrt{{{x}_{1}}^{2}+{{y}_{1}}^{2}}$ (if let *t* > 1, then we obtain a concentrator; or if let $0 < t < 1$, then we obtain a sparse device. To get an exact concentrator, here we set *t* = 2), and set *x*_1_ and *y*_1_ as unknowns. If let $trace({{\mu }_{1}}^{^{\prime} })=trace({{\mu }_{2}}^{^{\prime} })={\mu }_{a}+{\mu }_{b}$, we get the following parameters of the concentrator:

$${x}_{1}=0.0910,\,{y}_{1}=0.1771,\,\theta =-\,{27.20}^{\circ },$$

$${\mu ^{\prime} }_{1}=(\begin{array}{cc}0.5669 & -0.8924\\ -0.8924 & 3.1689\end{array}),{\mu ^{\prime} }_{2}=(\begin{array}{cc}2.4048 & -1.4828\\ -1.4828 & 1.3301\end{array}).$$

By setting *t* = 0.5, we can get a sparse device with the following parameters:

$${x}_{1}=0.1719,\,{y}_{1}=0.3822,\,\theta =-\,{24.22}^{\circ }$$

$${\mu ^{\prime} }_{1}=(\begin{array}{cc}3.3293 & -0.5918\\ -0.5918 & 0.4056\end{array}),{\mu ^{\prime} }_{2}=(\begin{array}{cc}3.2035 & 0.8381\\ 0.8381 & 0.5314\end{array}).$$

It is interesting to see that we only use one anisotropic material to design a rotator, a concentrator and a sparse device.

**Figure 4:**
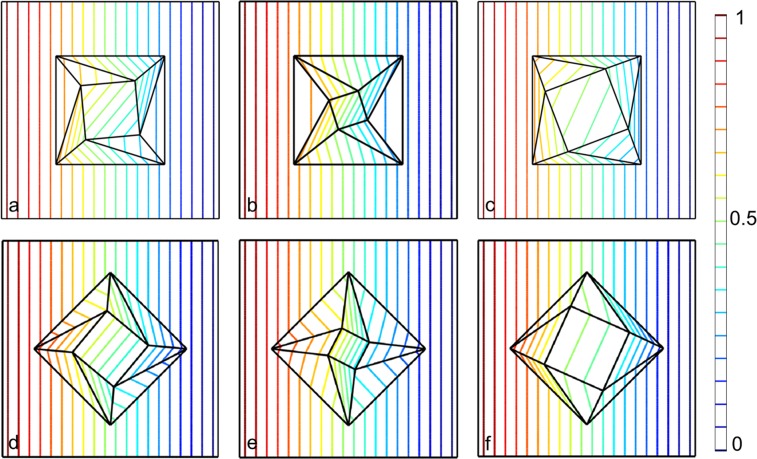
(**a**,**d**) Contour of the magnetic potential in and near rotator when the magnetic field is imposed from $\hat{x}$ direction and $\frac{\sqrt{2}}{2}\hat{x}+\frac{\sqrt{2}}{2}\hat{y}$ direction, respectively. (**b**,**e**) Contour of the magnetic potential in and near concentrator when the magnetic field is imposed from $\hat{x}$ direction and $\frac{\sqrt{2}}{2}\hat{x}+\frac{\sqrt{2}}{2}\hat{y}$ direction, respectively. (**c**,**f**) Contour of the magnetic potential in and near sparse device when the magnetic field is imposed from $\hat{x}$ direction and $\frac{\sqrt{2}}{2}\hat{x}+\frac{\sqrt{2}}{2}\hat{y}$ direction, respectively. All these devices above are composed of only one anisotropic material.

We use COMSOL Multiphysics to demonstrate the effectiveness of our strategy. In the simulation, we set the left boundary as magnetic potential with ${{\rm{A}}}_{z}={\rm{1}}\,{\rm{Wb}}/{\rm{m}}$, right boundary as magnetic potential with ${{\rm{A}}}_{z}={\rm{0}}\,{\rm{Wb}}/{\rm{m}}$, top and bottom boundaries as periodic conditions. To verify the omnidirectional performance of the devices, we also show the cases when the magnetic field source is rotated anticlockwise by 45°. Figure 4(a,d) show the contour of magnetic potential in and near rotator. One can see that the contour of the magnetic potential in the central region is rotated anticlockwise 39.85°, and that out of the rotator is undistorted. Figure 4(b,e) show the contour of magnetic potential in and near concentrator. It is obvious that the contour of magnetic potential in the central region is rotated anticlockwise 27.20°, and the density of contour of magnetic potential is double that out of the concentrator. Moreover, the contour out of the concentrator is undistorted. Figure 4(c,f) show the contour of magnetic potential in and near sparse device. One can see that the contour of magnetic potential in the central region is rotated anticlockwise 24.22°, and the density of the contour of magnetic potential is half that out of the sparse device. Moreover, the contour out of the sparse device is undistorted. Note that all these devices are composed of only one anisotropic material, which can be realized by the multilayered structure consisting of ferromagnetic materials and superconductor.

This strategy can also be applied to electrostatic field case and dc case. In dc case, we can use metals with different conductivity to design these devices mentioned above, for example, stainless iron, copper, and iron, which are used by Han *et al*. to design a cloak and a concentrator^36^.

## Conclusion

In this paper, a rotator consisting of homogeneous anisotropic materials is proposed, by applying the linear coordinate transformation. Our strategy is available in both time-harmonic electromagnetic field and static field. We further demonstrate that, for static field, only one anisotropic material is needed to design a rotator. In its central region, the density of the field can be altered to be denser or sparser, or stay the same. We show that the rotator for magnetostaic field can be realized by ferromagnetic materials and superconductor. For direct current, the rotator can be easily realized by metals with different conductivity, such as stainless iron, copper and iron. Both electromagnetic field case and static field case are numerically verified. The proposed method can be also extended to some new electromagnetic devices such as superscattering devices^37^, surface wave guided devices^38^, etc.
